# Methylomic Biomarkers of Lithium Response in Bipolar Disorder: A Proof of Transferability Study

**DOI:** 10.3390/ph15020133

**Published:** 2022-01-23

**Authors:** Cynthia Marie-Claire, Cindie Courtin, Frank Bellivier, Jan Scott, Bruno Etain

**Affiliations:** 1 INSERM UMR-S 1144, Optimisation Thérapeutique en Neurospsychopharmacologie (OTeN), Université de Paris, F-75006 Paris, France; cindie.courtin@parisdescartes.fr (C.C.); frank.bellivier@inserm.fr (F.B.); Bruno.Etain@inserm.fr (B.E.); 2 AP-HP, GH Saint-Louis—Lariboisière—F. Widal, Pole de Psychiatrie et de Médecine Addictologique, F-75475 Paris, France; 3 Fondation Fonda Mental, F-94000 Créteil, France; 4 Institute of Neuroscience, Newcastle University, Newcastle upon Tyne NE4 5PL, UK; jan.scott@newcastle.ac.uk

**Keywords:** DNA methylation, bipolar disorder, lithium, biomarkers, response, MS-HRM, validation, transferability, Alda scale

## Abstract

Response to lithium (Li) is highly variable in bipolar disorders (BD) and no clinical or biological predictors of long-term response have been validated to date. Using a genome-wide methylomic approach (SeqCapEpi), we previously identified seven differentially methylated regions (DMRs) that discriminated good from non-responders (prophylactic response phenotype defined using the “Alda” scale). This study is a proof of transferability from bench to bedside of this epigenetic signature. For this purpose, we used Methylation Specific High-Resolution Melting (MS-HRM), a PCR based method that can be implemented in any medical laboratory at low cost and with minimal equipment. In 23 individuals with BD, MS-HRM measures of three out of seven DMRs were technically feasible and consistencies between SeqCapEpi and MS-HRM-measures were moderate to high. In an extended sample of individuals with BD (*n* = 70), the three MS-HRM-measured DMRs mainly predicted nonresponse, with AUC between 0.70–0.80 according to different definitions of the phenotype (Alda- or machine-learning-based definitions). Classification tree analyses further suggested that the MS-HRM-measured DMRs correctly classified up to 84% of individuals as good or non-responders. This study suggested that epigenetic biomarkers, identified in a retrospective sample, accurately discriminate non-responders from responders to Li and may be transferrable to routine practice.

## 1. Introduction

Bipolar disorder (BD) is one of the leading causes of disability in young people [1,2]. BD is characterized by the recurrence of major depressive and (hypo)manic episodes interspersed by periods of remission or residual mood symptoms. Lithium (Li) is the first-line prophylactic treatment for BD and has proven efficacy for treating acute manic episodes, preventing mood relapses, and also for decreasing suicidal risk [3,4].

Unfortunately, response to Li is heterogeneous among cases with BD. After at least two consecutive years of treatment, only a fraction of patients receiving Li will display significant improvement in the frequency and/or severity of mood recurrences. In individuals with BD who received Li, three subpopulations (full or good responders (GR), partial responders (PaR) and non-responders (NR)) have been repeatedly identified, with around one third of the patients belonging to each group [5,6]. 

Considerable research efforts have been dedicated to the identification of clinical predictors of “good response” to Li. Several proposed predictors of good response to Li have been identified such as a “mania-depression-interval” sequence of episodes, absence of rapid cycling, absence of psychotic symptoms, family history of bipolar disorder, shorter pre-lithium illness duration, good social support and episodic evolution of BD [7,8]. However, no definitive clinical eligibility criteria for Li treatment can be reliably used for stratification and personalized approaches. 

In this context, the identification of biological markers that may be associated with the response to Li represents a mandatory first step towards a personalized medicine. The search for molecular markers of Li response in patients with BD is a very active field [9,10,11]. Promising results have been obtained in recent years using genetics with the identification of variants in two long noncoding RNAs [12] and in a total of 137 genes belonging to four pathways (muscarinic acetylcholine, Alzheimer’s amyloid-secretase, histamine H1R and G-protein-coupled receptor) associated with Li response [13]. Transcriptomic analyses in blood samples from 109 individuals with BD, before initiation of Li monotherapy and 2 or 8 weeks later, identified fourteen gene pathways involved in Li mechanism of action [14]. Expression levels of 15 miRNAs have been found to be associated with Li response in peripheral blood samples [15]. Telomere length has been associated with duration of Li treatment in responders [16,17,18]. Kinomic profiling identified 30 kinases differentially activated between responders and non-responders in lymphoblastoid cell lines [19]. However, several of these results have not been consistently replicated and some strategies would be complex to transfer from bench to bedside. 

Among biological markers, those related to epigenetic marks might prove to be particularly relevant in BD and for predicting treatment outcomes [20,21]. The investigation of DNA methylation applied to the response to Li in BD is very recent with most of the available studies having been published in the last 5 years [10,11]. Most of these studies were performed at the level of a single candidate gene. To address this gap in the literature, we recently published the first genome-wide analysis of DNA methylation profiles among patients with BD type 1 selected for their response to long-term treatment with Li [22]. By comparing good responders (GRs) to non-responders (NRs), we identified an epigenetic signature of response to Li that combines seven differentially methylated regions (DMRs). Further validation and transfer to the bedside of these biomarkers are now required. 

Genome-wide methylome approaches offer the advantage of exploring a very large number of CpG islands across the genome, mostly in terms of millions for the most recent developed approaches such as SeqCapEpi. However, these approaches generate a high cost per sample, are therefore mostly performed in small samples, and require massive computational resources to be analyzed. Given costs and requirements for equipment and analyses, these approaches may fail to be directly transferrable to the bedside. They mainly represent screening approaches that help defining the molecular targets of interest for a given research question. Once identified by these high-throughput approaches, specific targets are then identified by narrowing down the overall signal of interest to perform validation studies. The selection of a limited number of targets also offers the possibility to replicate in larger samples and to be transferred to bedside. For optimal transferability, these validation and transferable approaches should be reliable, usable in minimally-equipped laboratories and at low cost.

Methylation Specific High-Resolution Melting (MS-HRM) analysis is one possible approach for a transfer of an epigenetic signature to the bedside. MS-HRM is based on different melting temperatures (Tm) of methylated and unmethylated DNA [23]. After bisulfite conversion unmethylated cytosines are converted to uraciles and after PCR changed to thymines, while methylated cytosines are protected and remain unchanged. This change in base composition is able to differentiate between methylated and unmethylated DNA. Among the available DNA methylation validation methods, MS-HRM is one the easiest to implement, as well as cost and time effective [24]. The quantification calculations can be done easily and it therefore has greater potential for transferability from research laboratories to common laboratories practice than some other options.

The present study is a proof of transferability from bench to bedside of a methylomic signature that characterizes response to Li. This study is aimed at (1) validating using MS-HRM, the previously identified SeqCapEpi-derived signature of response to Li and (2) replicating results for the association with response to Li in an extended sample of individuals with BD type 1. 

## 2. Results

### 2.1. Optimization of the MS-HRM Tests

Two to five primers sets of HRM per DMR were designed to amplify the methylated and unmethylated DMRs. In the case of DMR57278, amplification was not detected. In the case of DMR101660 and DMR30347, Cq values were above 33 with all the primer pairs and therefore could not be measured. For DMR67206, the melt curves of the samples displayed a dome shape, indicating that the amplicons consisted of a group of PCR products with similar melt characteristics for methylated and unmethylated DNA and that they could therefore not be used for MS-HRM measures. 

**Figure 1:**
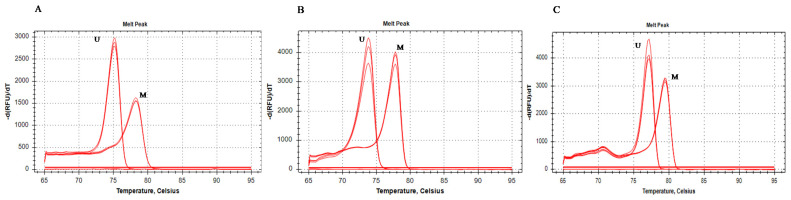
Raw melt curves (in triplicates) for 100% methylated (M) and 0% methylated (UM) bisulfite treated DNA standards. (**A**) DMR106540, (**B**) DMR24332, (**C**) DMR17107. Melt curve peaks had characteristically different shapes indicating that the methylated (M) and unmethylated (U) DNA are different products.

Technically satisfactory results were obtained for DMR24332, DMR17107 and DMR106540. The observed Tm for the 0% and 100% methylated DMRs are presented in Supplementary Table S1. The raw first derivative of the HRM curves obtained for the unmethylated and fully methylated DNA standards are presented in Figure 1. Peak-heights were used to generate the standard curves for determination of percent methylation for the three DMR analyzed. 

### 2.2. Consistency and Agreement between SeqCapEpi and MS-HRM DNA Methylation Measures (n = 23)

**Figure 2:**
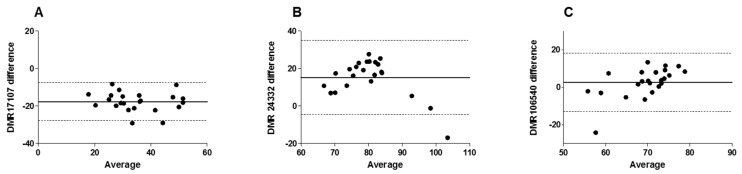
Nonparametric percentile method of Bland-Altman analysis. In the Bland–Altman plots shown above, the Y axis represents the difference between the measurements using the two methods and the X axis represents the average value of these measurements for (**A**) DMR17105, (**B**) DMR24332 and (**C**) DMR106540. The solid line represents the median of differences between measurements of the two methods and estimates of the systemic bias between the methods. The upper and the lower dashed lines represent limits of agreement (defined as the mean difference ± 1.96 SD of the difference between the two methods) between which 95% of measurements are situated.

In order to test consistency and agreement between the methylation percentages obtained with the SeqCapEpi method and the MS-HRM assays, we use individuals included in the previous published sample (*n* = 26), for whom DNA samples were still available (*n* = 23). First, the three MS-HRM assays for DMR24332, DMR17107 and DMR106540 were tested for equivalence between the two measures of methylation by SeqCapEpi (from previous experiment [22]) and MS-HRM methods using Bland-Altman plots (Figure 2). For the three DMRs, the majority of the dots are within the limits of agreement (i.e., ±1.96 SD). In addition, intraclass correlations (ICC) showed good consistency for DMR17107 (ICC = 0.87 95%CI (0.73–0.94); *p* < 0.001), and moderate consistency for DMR24332 (ICC = 0.51 95%CI (0.13–0.76); *p* = 0.006) and DMR106540 (ICC = 0.41 95%CI (0.004–0.70) *p* = 0.024). 

### 2.3. Performance of MS-HRM Assays in the Extended Sample (n = 70)

In order to measure the performance of the three DMRs to discriminate responders from non-responders to Li, we performed MS-HRM assays in an extended sample of BD type 1 patients, including also partial responders (only GRs and NRs were studied in the first sample). This extended sample comprised 70 individuals with a wide range of response to Li as shown in Table 1. Of note, 22 were also initially included in the previously published article, and one was not included due to lack of sufficient remaining DNA (Supplementary Figure S1). 

**Figure 3:**
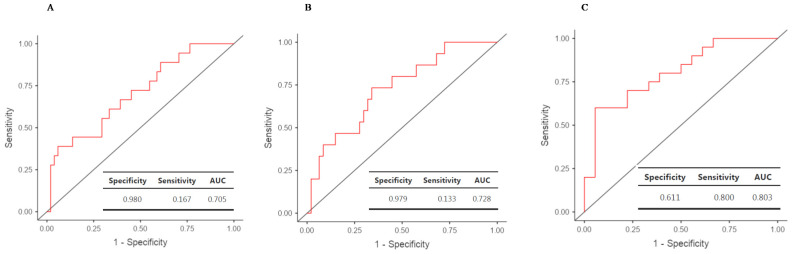
Receiver operating characteristics (ROC) curves showing the accuracy of the MS-HRM assay of the three DMRs to predict Li response using three different assessments of the response: (**A**) Alda-based broad definition, (**B**) Algo-based definition and (**C**) Alda-based narrow definition.

Logistic regression analyses were performed to estimate AUC and percentages of GRs and NRs correctly classified with the three DMRs and using three definitions of Li response: Alda-based broad definition (GR vs. NR/PaR—model 1, *n* = 70), Algo-based GR vs. others (model 2, *n* = 63) and Alda-based strict definition (GR vs. NR—model 3, *n* = 38) (Table 1). Clinical characteristics of all the samples identified as GR, PaR and NR according to the Alda scale are shown in Supplementary Table S2. Receiver operating characteristics (ROC) curves are presented in Figure 3. The lowest AUC (0.70 95% CI (0.57–0.84), *p* = 0.004) was obtained for the Alda-based broad definition (GR > 8 and NR < 8) with 98% of NR and 16.7% of GR being correctly classified. The performance of model 2 (Algo-based) was slightly improved (AUC = 0.73 95% CI (0.59–0.87), *p* = 0.002), with 97.9% of NR and 13.3% of GR being correctly classified. (Figure 3B). For the most stringent Alda-based definition of Li response (GR > 8 and NR < 3), the AUC was 0.80 95% CI (0.66–0.94) (*p* = 0.00002). This model correctly classified 80.0% of NR and 61.1% of GR, however with a reduction of the sample size since it did not take into account partial responders (Figure 3C).

**Figure 4:**
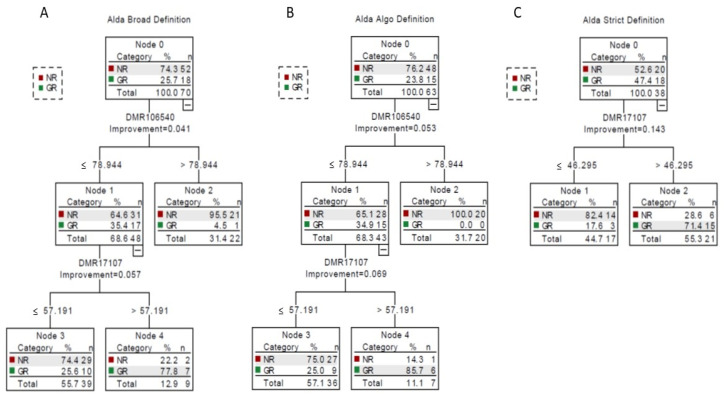
Classification tree models for Li response phenotypes (NR = nonresponse; GR = good response) and candidate DMRs. (A) Using model 1 (Alda-based broad definition), (**B**) model 2 (Algo-based definition), (**C**) model 3 (Alda-based narrow definition).

We then used Classification and Regression Tree (CRT) analyses to discriminate NRs and GRs based on the values of the three DMRs and the three definitions of the phenotype. For Alda-based broad definition, 81.4% of individuals were correctly classified (96.2% of NRs and 38.9% of GRs) based on values of two DMRs. Individuals in Node 2 (DMR106540 above 79) were mostly classified as NRs (95.5%). For individuals with low DMR106540 (below 79), further inclusion of DMR17707 led to a classification of 74.4% of individuals with low DMR17707 as NRs (Node 3), while 77.8% of individuals with high DMR17707 were GRs (Node 4) (see Figure 4A). The use of the Algo-based definition improved the classification to 84.1% of individuals being correctly classified (97.9% of NRs and 40.0% of GRs). All individuals with DMR106540 above 79 (Node 2) were NRs. 75% of individuals with a DMR106540 below 78 and a DMR17707 below 57 were NRs (Node 3), while 85.7% of individuals with a DMR106540 below 78 and a DMR17707 above 57 were GRs (Node 4) (see Figure 4B). Finally, the Alda-based narrow definition led to significantly different results with only one DMR being retained in the classification tree. 76.3% of individuals were correctly classified (70% of NRs and 83.3% of GRs). 82.4% of individuals with a DMR17107 below 46 (Node 1) were NRs, while 71.4% of individuals with DMR1707 above 46 were GRs (Node 2) (see Figure 4C). 

## 3. Discussion

In this study, we validated a methylomic signature of response to Li in patients with BD type 1. First, we develop a MS-HRM assay for three of the seven previously identified DMRs. Second, we examined the agreement of the results with the SeqCapEpi-obtained data in a similar sample, with reasonable consistencies between measures. Finally, we validated the performance of the MS-HRM assay to discriminate GRs from NRs in a larger sample of individuals with BD type 1. Using several definitions of Li response, we found that some DMRS might accurately predict nonresponse to Li.

The current study reports the use of MS-HRM, a PCR based method, as an approach to determine Li response using epigenetic-based biomarkers. Unlike most techniques used to analyze DNA methylation in research laboratories, this technique is cost-effective, easy to use and to implement in common medical analysis laboratories [25]. We showed that, in an extended sample (overlapping the one used in the first study), the data obtained with the MS-HRM assays are in good agreement with those obtained with the high-throughput method. Validation was further performed by extending the number of patients and increasing the heterogeneity in terms of response by including partial responders in the sample.

ROC curves analyses showed that this MS-HRM assay combining three DMRs can be relevant to characterize Li response in clinical samples from patients with BD type 1. Using machine-learning approaches to refine patients’ phenotyping of response to Li, and despite a small decrease of the sample size, we observed an improvement of the classification performances of the assay. This algorithm has the advantage of generating Li response phenotypes that are estimated according to the studied population. The use of the most stringent Alda-based definition of GR and NR yielded the best performance and accuracy to detect NR and GR when using AUC but not when using CRT. However, whilst excluding PaR enabled us to test models in this research study, the strategy is flawed with regard to transferability (we cannot utilize the findings if 30% or more of a clinical population is excluded). As a whole, the different models performed well when identifying NRs which is information that may be useful for clinicians (who may decide not to initiate Li in a given individual who is unlikely to respond).

Several limitations should be discussed. We were able to validate the first results obtained with another technique and extend the sample for the validation study. However, even if the extended sample of 70 is the largest published so far for epigenetic biomarkers of Li response, samples were partially overlapping and the extended sample cannot be considered as a pure replication sample. Second, the characterization of the phenotype was retrospective which does not allow us to determine if the observed methylation differences preexisted or were induced either by Li or others concomitant medications and/or by the disease state and/or the disease process which might differ between GRs and NRs. Third, these results may apply only to BD type 1, since we did not include any individuals with BD type 2 in the study. Finally, due to technical constraints, we were able to validate only three of the seven previously identified DMRs, and this approach might have excluded other DMRs of potential interest for classification. We cannot exclude that other DMRs might be relevant for increasing the classification of GRs and/or the performance of the MS-HRM assay.

This study provides a proof of transferability of epigenetic results obtained in research laboratories towards clinical practice settings. The proposed strategy should first be replicated in large, already recruited clinical samples with a retrospective assessment of the response to Li and second should be tested in prospective samples. This assay might also be used in combination with previously suggested clinical and sociodemographic predictors of response to Li, in order to maximize the prediction. Establishing biomarkers of Li response would represent an important breakthrough and a critical step towards precision medicine in bipolar disorder.

## 4. Methods

### 4.1. Sample

The samples consisted of French Caucasian individuals who met the DSM-IV criteria for BD type 1. Patients were recruited from one university-affiliated psychiatric department in France (Paris). Patient inclusion criteria has been described previously [26]. This study was approved by the French medical ethics committee (Comité de Protection des Personnes (CPP)–IDRCB2008_AO1465_50 VI–Pitié-Salpêtrière 118-08) and carried out according to the approved guidelines. This study is a secondary analysis of the research protocol registered under the number NCT02627404 in ClinicalTrials.gov (accessed on 20 November 2021). The current study includes individuals with a confirmed diagnosis of BD-I who were prescribed Li and whose response was assessed using the Alda scale.

### 4.2. Phenotyping of Response to Lithium

#### 4.2.1. Original Alda Scale

The response to Li was rated using the “Retrospective Criteria of Long-Term Treatment Response in Research Subjects with Bipolar Disorder”, also referred to as the “Alda scale” [27]. This scale was specifically developed to allow a retrospective assessment of prophylactic response to treatment in naturalistic conditions. In accordance with the available literature [5], patients with total score ≥ 8 were characterized as good responders (GR) and patients with total score ≤ 3 were characterized as non-responders (NR). Remaining individuals were classified as partial responders (PaR). In model 1 (Alda-based broad definition), the phenotypes were as follow: GR (total score ≥ 8) vs. NR + PaR (total score < 8) to maximize the number of patients in the analysis. In model 3 (Alda-based narrow definition), the phenotypes were as follow: GR (total score ≥ 8) vs. NR only (total score ≤ 3); this model reduces the sample size (by excluding PaR) but prevents the misclassification of PaR to maximize the contrast between groups. Model 2 (Algo-based definition) is based on a machine learning approach to classified GR and NR as described below.

#### 4.2.2. Machine Learning Approach to Rating the Alda Scale

As described in [28], a machine learning algorithm was used with a set of “if-then” rules to determine the probability of GR and NR. The sequence to enter the B scale item scores is treatment complexity (adherence and polypharmacy), then duration of Li treatment, and/or illness activity (the exact sequence and combination of item scores is generated by the machine learning model). When the optimal classification is reached, the algorithm stops running irrespective of whether all B items have been included (for details see [28]). Here, we report the findings on Li response phenotypes as a categorical measure (GR vs. others; model 2). This alternative approach of using the Alda scale has been recently proposed to produce an estimate of Li response while improving the performance of the Alda scale [28]. This classification has been successfully used to identify clinical characteristics and markers associated with Li response [29,30].

### 4.3. DNA Isolation and Bisulfite Modification

DNA was isolated from total peripheral blood collected at inclusion. Native genomic DNA was extracted using standard procedures and stored at −20 °C until use. The genomic DNA input was 200 ng to be modified with sodium bisulfite using the EZ DNA™ methylation kit (Zymo Research, Irvine, CA, USA). Human methylated and unmethylated DNA standards were prepared by diluting 200 ng of fully methylated DNA by 200 ng of fully unmethylated DNA before bisulfite conversion in 0, 10, 20, 30, 40, 50, 60, 70, 80, 90 and 100% methylated to unmethylated template ratios. Bisulfite modified DNA was eluted in 10 μL of nuclease-free water according to the manufacturer’s instructions. The modified DNA was quantified with a NanoDrop One Spectrophotometer (Ozyme, Saint-Cyr-l’École, France).

### 4.4. Methylation Sensitive High-Resolution Melting

Methylation-Sensitive High-Resolution Melting (MS-HRM) was performed on a CFX384 Touch Real-Time PCR Detection System (Biorad Laboratories, Des Plaines, IL, USA). Primers were designed according to the principles outlined by Wojdacz and colleagues [31,32] using the Bisearch online tool (http://bisearch.enzim.hu/; accessed on 22 June 2020). PCR reactions were performed in a final volume of 10 μL, containing 200 nM of each primer, 5 μL of Precision Melt Supermix (Biorad Laboratories) and 10 ng of bisulfite-treated DNA. The initial denaturation (95 °C, 3 min) was followed by 45 cycles of 10 s at 95 °C, 30 s at 50 °C, 30 s at 72 °C. The HRM step consisted of a denaturation of all products at 95 °C for 30 s followed by an annealing at 60 °C for 1 min. Samples were then slowly warmed to 95 °C at 0.2 °C per second, holding for 10 s after each stepwise increment and fluorescence data were collected. The annealing temperature was chosen to obtain a near-proportional amplification of methylated and unmethylated templates and each sample was analyzed in triplicate.

For the DNA methylation assessment, as described above, bisulfite converted dilutions of methylated and unmethylated DNA standards were analyzed together with the samples. Peak-heights were calculated automatically with the CFX Maestro Software (Version 2.2; Bio-Rad Laboratories, Inc., Hercules, CA, USA). Linear curves of the peak-heights of the Tm first derivative of HRM curves against the methylation percentage of the standard were plotted [33].

### 4.5. Statistical Analysis

Statistical analyses were performed using Jamovi software (Version 1.6; https://www.jamovi.org/; accessed on 11 September 2021) and SPSS (Version 27; Armonk, NY, USA). Bland-Altman plots were used to assess agreement between the measure of DNA methylation with the SeqCapEpi and MS-HRM methods. We calculated the mean difference of the two measurements and corresponding SDs. Bland-Altman plots were created by plotting the means of the SeqCapEpi and MS-HRM values against difference scores between the two measures. This allows the assessment of whether one method consistently under- or overestimates measurements of the same variable as compared to the other method. Intraclass correlations (ICC) between measures were also calculated.

To estimate the discriminatory power, sensitivity, and specificity for the combination of the 3 tested DMRs, logistic regression analyses, followed by receiver operating characteristic (ROC) curve analysis were performed for the three definitions of the phenotype. Finally, for the three definitions of the phenotype, we employed a decision approach to classify GR and NR, according to the three DMRS and based on classification and regression trees (CRT). CRT is a complete binary tree algorithm that creates a tree-based model. It classifies cases into groups (GR versus NR) and predicts values of a dependent variable based on values of predictor variables (DMRs). In the figures shown, the order of importance of explanatory variables (and the cut-off values) is explicitly represented by the tree structure. The model starts with the root node which contains all cases. We use the following criteria for building the tree (parent node *n* = 10, child node *n* = 5). Trees were pruned to avoid overfitting.

## 5. Conclusions

In conclusion, using a method that can be transferrable to routine practice, our results suggest that epigenetic biomarkers, identified in a retrospective sample, accurately discriminate non-responders from responders to Li. The proposed assay could be used in combination with previously suggested clinical and socio-demographic predictors of response to Li, in order to maximize the prediction.

## Figures and Tables

**Table 1:** Clinical characteristics of all the patients with bipolar disorder type 1 included in the extended validation sample and GR/NR distribution in the three models used to define Li response.

	*n* (%)	Median IQR
*n* *	70	
Alda total score		6 (3–8)
Alda-based GR/PaR/NR	18/32/20	
Sex ratio Male/Female	33/37	
Age		43 (35–53)
BMI		24.5 (22.5–27.6)
Smokers yes/no	33/35	
Li current use yes/no	63/7	
Model 1 GR ≥ 8/NR ≤ 7 (Alda-based broad definition)	18/52	
Model 2 GR/NR (Algo-based)	15/48	
Model 3 GR ≥ 8/NR ≤ 3 (Alda-based strict definition)	18/20	

Li: Lithium, GR: good responder, PaR: partial responder, NR: nonresponder, BMI: body mass index, BD: bipolar disorder, *n*: number. * 22/70 individuals were used in the previously published article.

## Data Availability

Due to an on-going patent application, the data are not available for individuals who are not investigators of the study. The data are not publicly available also due to ethical restrictions (e.g., participants have not given approval for the dataset to be shared with other research groups, etc.).

## Supplementary Materials

The following are available online at https://www.mdpi.com/article/10.3390/ph15020133/s1, Table S1: MS-HRM amplicons characteristics for the three validated DMRs; Table S2: Clinical characteristics of all the patients with bipolar disorder type 1 included in the extended validation sample; Figure S1: Summary of sample overlaps in the SeqcapEpi and MSH-RM experiments. (Total number = 74, with 70 being included in the MSH-RM experiment).
