# Supplementary material for: Methylomic Biomarkers of Lithium Response in Bipolar Disorder: A Proof of Transferability Study
Source: Pharmaceuticals (Basel). 2022 Jan 23;15(2):133. doi: 10.3390/ph15020133 (PMC8877131; doi:10.3390/ph15020133)
Supplement: Supplementary file 1 [file pharmaceuticals-15-00133-s001.zip › Supl Tables.pdf]

DMR ID	length (bp)	Tm 0% methylated	Tm 100% methylated
17107	169	77.20	79.40
106540	116	75.20	78.20
24332	147	73.80	77.80

Supplementary Table S1: MS-HRM amplicons characteristics for the three validated DMRs.

DMR: Differentially Methylated Regions; ID: Identity; bp: base pair; Tm: melting temperatures*; MS-HRM: Methylation Specific High-Resolution Melting*

	GR	PaR	NR
N*	18	32	20
Alda Range	8-10	4-7	0-3
Ratio male/female	10/8	13/19	10/10
Age median (IQR)	41.1 (33-49.2)	42.9 (35.0-53.9)	42.8 (41.3-50.7)
BMI median (IQR)	25.6 (23.1-27.1)	24.3 (22.6-27.9)	24.5 (22.6-28.4)
Smokers yes/no	7/10	15/16	11/9
Current medications			
Lithium (yes/no)	17/1	32/0	14/6
Anticonvulsants (yes/no)	2/16	15/16	9/11
Atypical antipsychotics (yes/no)	1/17	13/19	12/8
Antidepressants (yes/no)	1/17	10/21	7/13

Supplementary Table S2: Clinical characteristics of all the patients with bipolar disorder type 1 included in the extended validation sample.

*GR: good responder, PaR: partial responder, NR: non responder according to the Alda scale; BMI: body mass index; IQR: Interquartile; BD: bipolar disorder, N: number. * 22/70 individuals were used in the previously published article.*
