# Supplementary material for: Methylomic Biomarkers of Lithium Response in Bipolar Disorder: A Proof of Transferability Study
Source: Pharmaceuticals (Basel). 2022 Jan 23;15(2):133. doi: 10.3390/ph15020133 (PMC8877131; doi:10.3390/ph15020133)
Supplement: Supplementary file 1 [file pharmaceuticals-15-00133-s001.zip › Supplementary Figure 1.pdf]

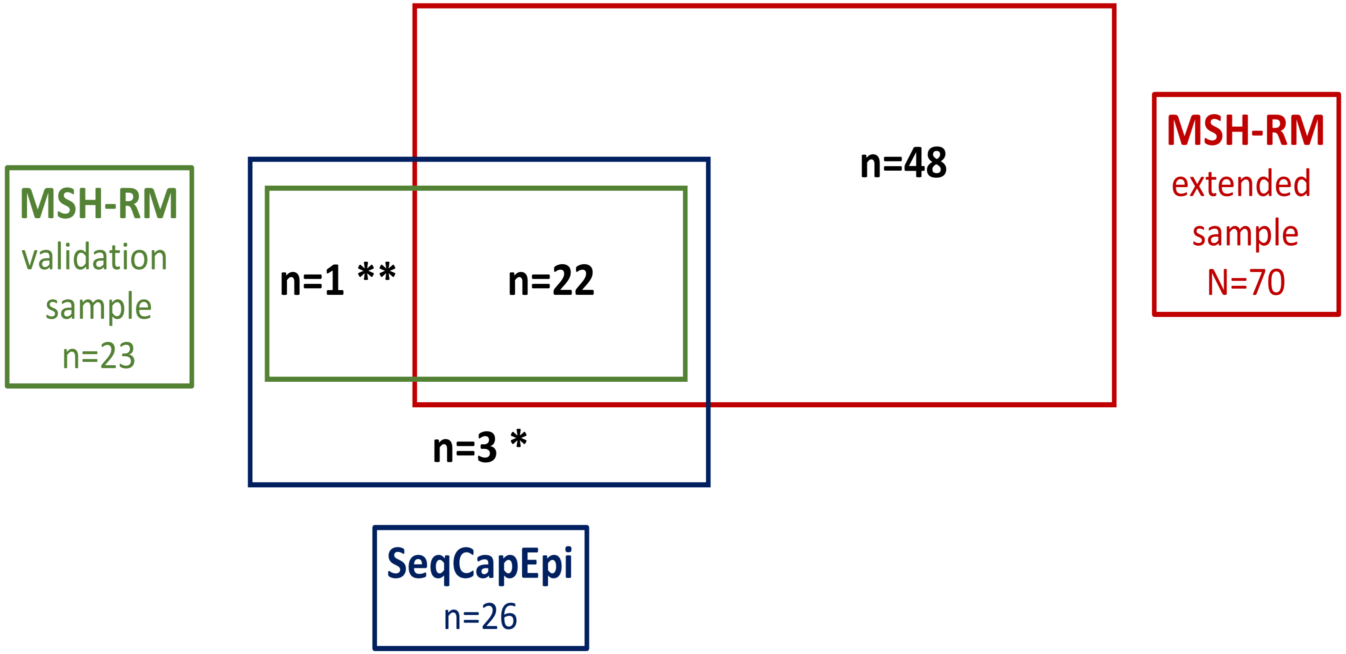

* 3 individuals with not enough DNA to be included in the MSH-RM validation sample after SeqCapEpi

* * 1 individual with not enough DNA to be included in the MSH-RM extended sample after the MSH-RM validation

Supplementary Figure S1: Summary of sample overlaps in the SeqcapEpi and MSH-RM experiments. (Total number = 71).
